# Impact of 18F-DCFPyL-PSMA PET/CT on Radiotherapy Planning and Treatment Intensification in Occult Biochemical Recurrence of Prostate Cancer After Radical Prostatectomy

**DOI:** 10.3390/cancers18182919

**Published:** 2026-09-09

**Authors:** Francesco Amorelli, Pedro Plaza, Augusto Natali, Juan Sebastian Blanco, Xavier Duran, Palmira Foro

**Affiliations:** 1 Department of Radiation Oncology, Catalan Institute of Oncology (ICO), Hospitalet de Llobregat, 08908 Barcelona, Spain; 2 Ph.D. Program, Doctoral School, Pompeu Fabra University, 08002 Barcelona, Spain; pforo@psmar.cat; 3 Department of Nuclear Medicine, Hospital del Mar, 08003 Barcelona, Spain; pedrojose.plaza.lopez@hmar.cat (P.P.); juansebastian.blanco.cano@hmar.cat (J.S.B.); 4 Department of Laboratory Medicine, CLILAB Diagnostics, Hospitalet de Llobregat, 08906 Barcelona, Spain; anatali@cli.cat; 5 Medical Statistics Unit (MSU) MARData, Hospital del Mar, 08003 Barcelona, Spain; xduran@researchmar.net; 6 Department of Radiation Oncology, Hospital del Mar, 08003 Barcelona, Spain

**Keywords:** prostate cancer, occult biochemical recurrence, radical prostatectomy, radiotherapy planning, 18F-DCFPyL PET/CT, detection rate, biochemical response

## Abstract

Biochemical recurrence (BCR) after radical prostatectomy (RP) frequently occurs despite negative conventional imaging, making salvage treatment decisions challenging. We evaluated the impact of 18F-DCFPyL-PSMA PET/CT on disease detection and radiotherapy planning in patients with occult BCR. Methods: We conducted a prospective single-center observational study including 85 patients with BCR after RP, negative or inconclusive conventional imaging, and PSA levels between 0.2 and 1.0 ng/mL or persistent PSA after previous local treatment. Detection rates (DRs) were evaluated according to PSA level, PSA doubling time (PSA-DT), and International Society of Urological Pathology (ISUP) grade. Changes in radiotherapy planning and treatment intensification were assessed by comparing intended management before and after PSMA PET/CT. Results: PSMA PET/CT detected recurrent disease in 53% of patients. DRs increased significantly with PSA level, reaching 31.3% for PSA 0.2–0.5 ng/mL, 60.0% for PSA 0.5–0.9 ng/mL, and 77.8% for PSA 1.0 ng/mL (*p* < 0.001). Detection was also associated with shorter PSA-DT and higher ISUP grade. Pelvic lymph nodes (LNs) represented the predominant site of recurrence, accounting for 48 of 79 detected lesions. Treatment strategy changed in 97.8% of patients with positive scans, while radiotherapy planning was modified in 84.4%. PSMA PET/CT enabled expansion of target volumes, focal dose escalation, metastasis-directed stereotactic body radiotherapy (SBRT), systemic treatment intensification, and in selected patients with negative findings, treatment de-escalation to active surveillance. Following PSMA-guided treatment adaptation, the median PSA level at 24 months was 0.08 ng/mL, with early biochemical responses (BRs) observed during follow-up. Conclusions: 18F-DCFPyL-PSMA PET/CT provides clinically meaningful information beyond disease detection by substantially modifying radiotherapy planning and treatment selection in patients with occult BCR after RP. These findings support the integration of molecular imaging into salvage treatment planning to enable more personalized, biology-driven radiotherapy strategies.

## Simple Summary

Patients with prostate cancer (PC) may experience a rise in prostate-specific antigen (PSA) after radical prostatectomy (RP) despite having no detectable disease on conventional imaging. In this situation, treatment decisions are often based on clinical risk factors rather than the true location of recurrent disease. We evaluated whether 18F-DCFPyL prostate-specific membrane antigen (PSMA) positron emission tomography/computed tomography (PET/CT) could better identify recurrent cancer and improve radiotherapy planning. In our prospective study, this imaging technique detected previously occult disease in more than half of the patients and led to substantial modifications in treatment strategy, including adaptation of radiotherapy target volumes, dose escalation, metastasis-directed stereotactic radiotherapy, systemic treatment intensification, or, in selected patients with negative scans, treatment de-escalation. These findings demonstrate that molecular imaging can support more personalized and precise salvage treatment, helping clinicians tailor radiotherapy according to the actual extent of recurrent disease rather than relying solely on conventional imaging or clinical risk factors.

## 1. Introduction

Biochemical recurrence (BCR) after radical prostatectomy (RP) occurs in approximately 20–50% of patients with prostate cancer (PC) [1,2,3]. BCR is typically defined by a PSA level exceeding 0.2 ng/mL [4]. In this setting, salvage radiotherapy (SRT), typically delivered to the prostate bed (PB), represents the standard curative approach [5,6,7], achieving 5-year biochemical control rates of approximately 50–60% [8,9]. However, treatment failure remains frequent and is often attributed to the presence of disease outside the PB, including pelvic lymph nodes (LNs) or distant metastases, which are not adequately detected with conventional imaging [10,11]. Conventional imaging modalities, including contrast-enhanced computed tomography (CT), pelvic magnetic resonance imaging (MRI), and bone scintigraphy, have well-recognized limitations, particularly at low PSA levels [12,13], where sensitivity for detecting early local and systemic recurrence is reduced and false-negative findings are frequent, thereby complicating both diagnostic evaluation and treatment decision-making [14,15]. Consequently, patient management is often guided by international consensus guidelines [16,17,18].

Presently, there is no clear consensus on the optimal imaging modality for patients with BCR, particularly at low PSA levels [19,20]. Although positron emission tomography/computed tomography (PET/CT) has demonstrated superior sensitivity compared with conventional imaging and is increasingly used in this setting [21], earlier radiotracers such as choline and fluciclovine, although associated with improved detection rates (DRs), still show suboptimal diagnostic performance in early BCR [22,23,24,25,26]. Against this background, PET/CT targeting prostate-specific membrane antigen (PSMA) has emerged as a highly sensitive imaging modality [27]. PSMA is a transmembrane glycoprotein overexpressed in most PC cells, with expression levels correlating with tumor aggressiveness and metastatic potential [28,29].

PSMA ligands, including 18F-DCFPyL, 18F-PSMA-1007, and 68Ga-PSMA-11, bind with high affinity to the extracellular domain of PSMA and have demonstrated high diagnostic performance in detecting disease across different stages, with tracer uptake correlating with tumor aggressiveness [30,31,32,33].

Among these, 18F-labeled agents such as 18F-DCFPyL offer several advantages over earlier compounds [34,35], including improved spatial resolution, higher tumor-to-background ratios, and greater production scalability, resulting in superior sensitivity and specificity for detecting both local and distant recurrence compared with conventional imaging [36,37]. Accordingly, PSMA PET/CT is now recommended in international guidelines for the evaluation of BCR, although the optimal PSA threshold for its use remains to be clearly defined [38,39,40].

Beyond its diagnostic performance, this imaging modality plays an increasingly important role in guiding therapeutic decision-making [41,42,43]. Although several studies have demonstrated management changes in a substantial proportion of patients with BCR, the nature and determinants of these changes remain incompletely understood, particularly in the setting of early recurrence and negative conventional imaging. Moreover, it remains uncertain whether the increased sensitivity of PSMA PET translates into more individualized and biologically driven radiotherapy strategies. In current practice, SRT is often delivered empirically to the PB, with or without elective pelvic nodal irradiation (EPRT). However, PSMA PET/CT frequently reveals occult nodal or distant disease not detected on standard imaging. This may lead to significant modifications in treatment strategy, including expansion or modification of radiotherapy volumes, focal dose escalation, or a shift toward metastasis-directed therapy or systemic treatment [44,45,46].

The aim of this study was to evaluate the impact of 18F-DCFPyL-PSMA PET/CT on salvage radiotherapy planning in patients with occult BCR after RP and negative conventional imaging. Specifically, we aimed to quantify and characterize imaging-guided modifications in radiotherapy planning, with a particular focus on changes driven by positive findings.

## 2. Materials and Methods

This prospective observational study was conducted at a single institution in Barcelona, Spain. The study population comprised 85 patients evaluated between September 2021 and January 2024 for occult BCR following RP. Eligible patients included those with PSA levels of 0.2–1.0 ng/mL who were being considered for SRT and those with persistently detectable PSA after previous ART or salvage irradiation of the PB. These clinical scenarios were intentionally included to reflect the real-world spectrum of patients with occult BCR after RP who were referred for PSMA PET/CT evaluation at our institution. Inclusion additionally required the absence of definite local or distant recurrence on conventional imaging, including contrast-enhanced CT, pelvic MRI, and bone scintigraphy. Before 18F-DCFPyL-PSMA PET/CT acquisition, clinical and pathological information was prospectively recorded, including the surgical procedure, Gleason score, ISUP grade group, TNM stage, previous ADT exposure, serum PSA level (ng/mL), and PSA-DT in months.

### 2.1. Imaging Protocol and Image Interpretation

All 18F-DCFPyL-PSMA PET/CT examinations were performed in accordance with the institution’s standardized imaging protocol. Each patient received an intravenous dose of 299–333 MBq of 18F-DCFPyL, manufactured under good manufacturing practice conditions and supplied by Curium Pharma (Alcobendas, Spain). Whole-body PET/CT images were acquired 90 min after tracer administration using a Siemens Biograph mCT 40 scanner (Siemens Healthineers, Erlangen, Germany), in accordance with the recommendations of the joint EANM–SNMMI procedure guideline [31]. Intravenous iodinated contrast material was administered unless contraindicated. Furosemide was not routinely administered. When clinically indicated, an additional 5 min delayed pelvic PET was obtained. The 18F-DCFPyL-PSMA PET/CT images were independently reviewed using SyngoVia software (VB80D, Siemens Healthineers, Erlangen, Germany) by two experienced nuclear medicine physicians. Foci of abnormal radiotracer uptake that could not be attributed to physiological tracer distribution were considered positive when classified as consistent with or suggestive of PC according to the PROMISE criteria [47,48] and as PSMA-RADS 4 or 5 according to the Prostate-Specific Membrane Antigen Reporting and Data System [49]. Disagreements between readers were resolved by consensus. Positive findings were categorized according to their anatomical distribution as local recurrence (LR), pelvic LN involvement, extra-pelvic LN involvement, bone metastases, or visceral disease. The nuclear medicine physicians involved in image interpretation had more than 10 years of experience in PET/CT imaging of PC, including extensive experience with choline-based radiotracers and with three different PSMA ligand-targeted radiotracers since 2018.

We evaluated the per-patient disease DR of 18F-DCFPyL-PSMA PET/CT according to lesion location and examined its associations with relevant clinical and biochemical variables, including PSA level (0.2–0.5, 0.5–0.9, and 1.0 ng/mL), PSA-DT (<6, 6–12, and >12 months), and ISUP grade group. Exploratory receiver operating characteristic curve analyses were performed to assess the discrimination of PSA and PSA-DT for PET positivity.

### 2.2. Treatment Intensification and Changes in Radiotherapy Planning

We evaluated potential changes in radiotherapy planning and treatment intensification by comparing the original pre-PSMA treatment intention with the post-PSMA treatment intention. The original treatment strategy was prospectively defined and documented prior to 18F-DCFPyL-PSMA PET/CT. Each patient was initially evaluated at the Radiation Oncology clinic, and the case was subsequently discussed by the multidisciplinary tumor board. The intended treatment strategy was defined according to clinical and pathological characteristics, previous treatment exposure, and conventional imaging findings. For patients without prior radiotherapy, SRT to the PB was planned at a total dose of 66–68 Gy in 33–34 fractions (2 Gy per fraction). In selected cases, EPRT to 46–48 Gy was considered based on established risk nomograms (Roach formula >20%) [50]. Additionally, ADT was recommended in the presence of high-risk features, including PSA > 0.7 ng/mL, ISUP grade > 2, and life expectancy > 10 years [51]. For patients who had previously received ART or SRT, management options included the addition of ADT (if not previously administered) or active surveillance with close monitoring.

Following 18F-DCFPyL-PSMA PET/CT, treatment strategies were reassessed through multidisciplinary discussion, and modifications to the initial intent were defined and documented according to PET findings (post-PSMA intention). Changes in management were categorized according to their clinical impact as no change, minor changes, or major changes. Minor changes were defined as those in which treatment intent was maintained, with adjustments to radiotherapy planning, including expansion of target volumes to incorporate pelvic radiotherapy (PRT) and dose escalation to PSMA-positive pelvic LNs or LR within the PB, with or without the addition of ADT. Major changes were defined as changes in treatment intent driven by the identification of oligometastatic disease, including a shift toward SBRT targeting PSMA-positive lesions, with or without systemic treatment intensification using ADT and androgen receptor signaling inhibitors (ARSI), or a recommendation to defer SRT in favor of active surveillance with close monitoring.

Following treatment adaptation, PSA levels were assessed longitudinally for up to 24 months. BR was defined as a reduction of ≥50% from the pretreatment PSA level, consistent with the definition used in the evidence summarized in the EAU guidelines regarding BR following PET-guided treatment [40]. Time to biochemical response was categorized according to the first follow-up assessment at which this reduction was observed: ≤3 months, >3 to 6 months, or >6 months. Patients who did not achieve this reduction or who subsequently experienced biochemical progression were recorded separately.

### 2.3. Statistical Analysis

Continuous variables are summarized using means and standard deviations or medians and interquartile ranges, as appropriate, while categorical variables are reported as frequencies and percentages. Between-group comparisons according to PSMA PET/CT results (PET-negative vs. PET-positive) were performed using the chi-squared test or Fisher’s exact test for categorical variables and the Mann–Whitney U test for continuous variables. The non-parametric test was selected because several continuous variables did not satisfy the assumption of normality. To explore the potential influence of previous treatment exposure, an additional exploratory subgroup analysis was performed according to prior radiotherapy and ADT use, comparing PSMA PET/CT positivity between groups using Fisher’s exact test. Cohort-specific cutoff values for PSA (ng/mL) and PSA-DT were determined using ROC curve analysis. Associations between pre-PET clinical variables and PSMA PET/CT positivity were considered exploratory and were not interpreted as evidence of independent predictive effects. A priori sample size estimation was conducted assuming an expected proportion of management change of 80%, with a two-sided 95% confidence level and a margin of error of 10%, yielding a required sample size of approximately 62 patients. The final sample size exceeded this requirement.

All statistical analyses were performed using Stata version 15.1, and a *p*-value <0.05 was considered statistically significant.

## 3. Results

A total of 85 patients were included in the study. Baseline characteristics are summarized in Table 1. The median age was 69 years (range 48–74). At diagnosis, pathological stages were distributed as T2a–b (8.2%), T2c (42.4%), T3a (24.7%), T3b (23.5%), and T4 (1.2%). Nodal status was N0 in 57.6%, Nx in 37.6%, and N1 in 4.7% of cases, while 62.4% were classified as M0 and 37.6% as Mx. Regarding tumor characteristics, 43.5% of patients had a Gleason score < 8 and 56.5% ≥ 8, with a mean PSA at diagnosis of 11.24 ng/mL. All patients underwent RP, with pelvic lymph node dissection (PLND) performed in 51.8% of cases. Prior radiotherapy had been delivered in 52.9% of patients, including ART in 14.1% and SRT in 38.8%. ADT had been administered in 38.8% of cases.

### 3.1. 18F-DCFPyL-PSMA PET/CT Detection Rate

PSMA PET/CT was positive in 53% of patients (45/85). Before imaging, the median PSA level was 0.59 ng/mL (IQR, 0.29–1.00), and the mean PSA-DT was 7 months (range, 3–17.1 months). Compared with PET-negative patients, PET-positive patients had significantly higher PSA levels and shorter PSA-DT (*p* = 0.004 and *p* = 0.005, respectively). Among patients with positive PET findings, 54% had a Gleason score ≥ 8, and 28.9% were classified as ISUP grade group 3 and 57.7% as ISUP grade groups 4–5. Additionally, 15.6% of PET-positive patients had pN1 disease at diagnosis. The distribution of pathological T stages (T2c–T3b) was comparable between the PET-positive and PET-negative groups.

Furthermore, a subgroup analysis was performed according to previous treatment exposure. PSMA PET/CT positivity was observed in 42.5% of patients without prior RT and in 62.2% of previously irradiated patients, although this difference did not reach statistical significance (*p* = 0.084). Similarly, PET positivity was 63.6% among patients with prior ADT exposure, also without reaching statistical significance (*p* = 0.083).

The disease DR increased progressively with higher PSA levels, reaching 31.3%, 60%, and 77.8% for PSA 0.2–0.5, 0.5–0.9, and 1.0 ng/mL, respectively (*p* < 0.001). Across PSA strata, nodal involvement was the most frequent site of disease, particularly at higher PSA levels (34.2% and 44.4% for PSA 0.5–0.9 and 1.0 ng/mL, respectively). Bone metastases were also observed in patients with PSA 0.2–0.5 (15.6%) and 0.5–1.0 ng/mL (16.7%), while visceral involvement was uncommon and limited to higher PSA levels. LR in the PB was less frequently detected across all PSA groups.

**Figure 1:**
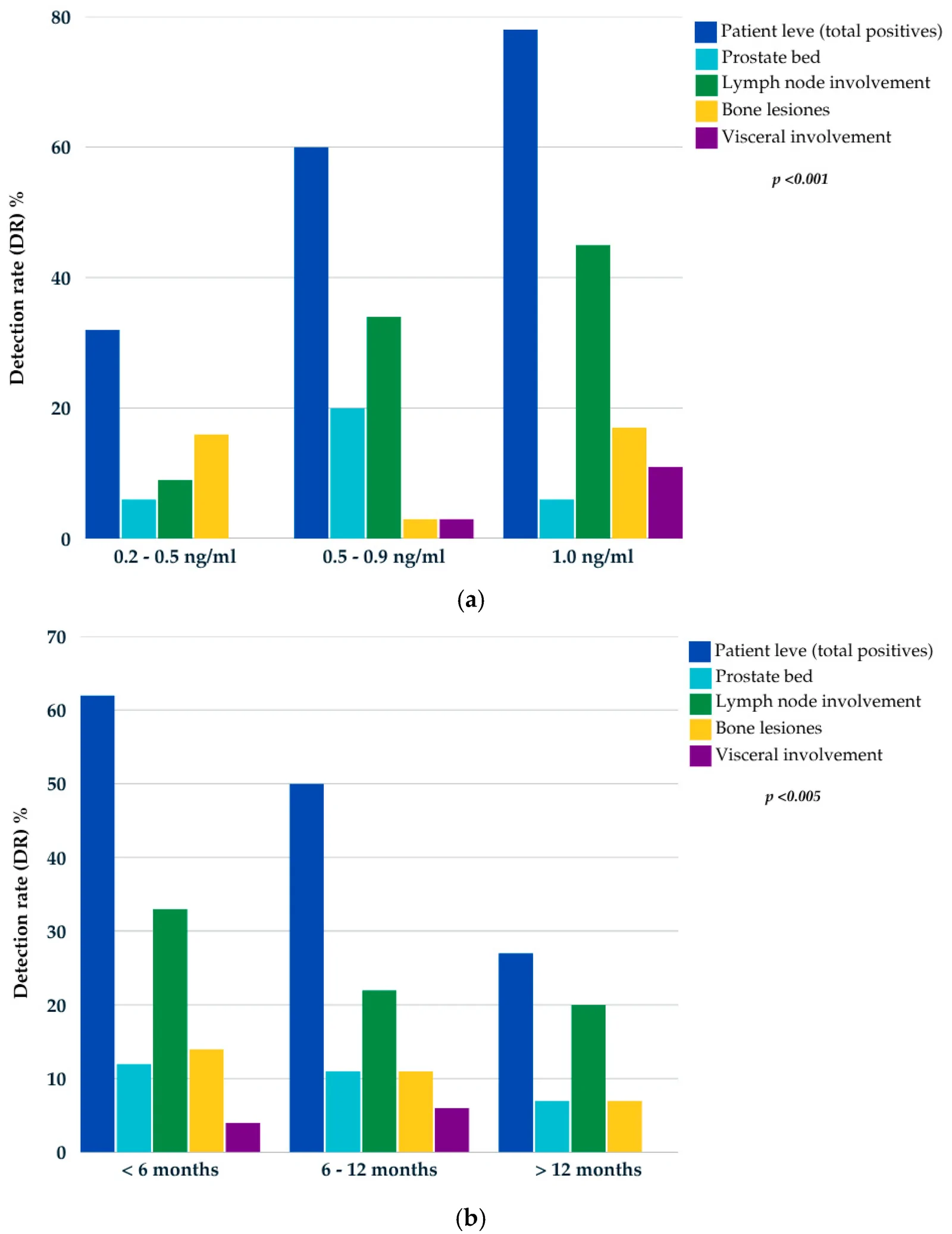
Detection rates with 18F-DCFPyL-PSMA PET/CT according to lesion location: (**a**) PSA level (ng/mL) and (**b**) PSA-DT (months).

DR also varied according to PSA-DT, with higher DR observed in patients with shorter PSA-DT (*p* < 0.005). Specifically, DRs were 61.5%, 50%, and 26.7% for PSA-DT < 6, 6–12, and >12 months, respectively. LN involvement remained the predominant pattern of disease, particularly in patients with rapid PSA kinetics (<6 months, 32.8%), while bone and visceral metastases were more frequently observed in this group (Figure 1).

Similarly, DRs increased with higher-ISUP-risk groups, with DRs of 24%, 59%, and 68.4% for ISUP 1–2, 3, and 4–5, respectively (*p* < 0.02), further supporting the association between tumor aggressiveness and PSMA PET positivity.

In our cohort, a total of 79 lesions were identified among patients with positive 18F-DCFPyL-PSMA PET/CT, including 10 LR in the PB, 48 pathological LNs, 18 bone lesions, and three visceral lesions, confirming the heterogeneous distribution of recurrent disease and the predominance of nodal involvement.

**Figure 2:**
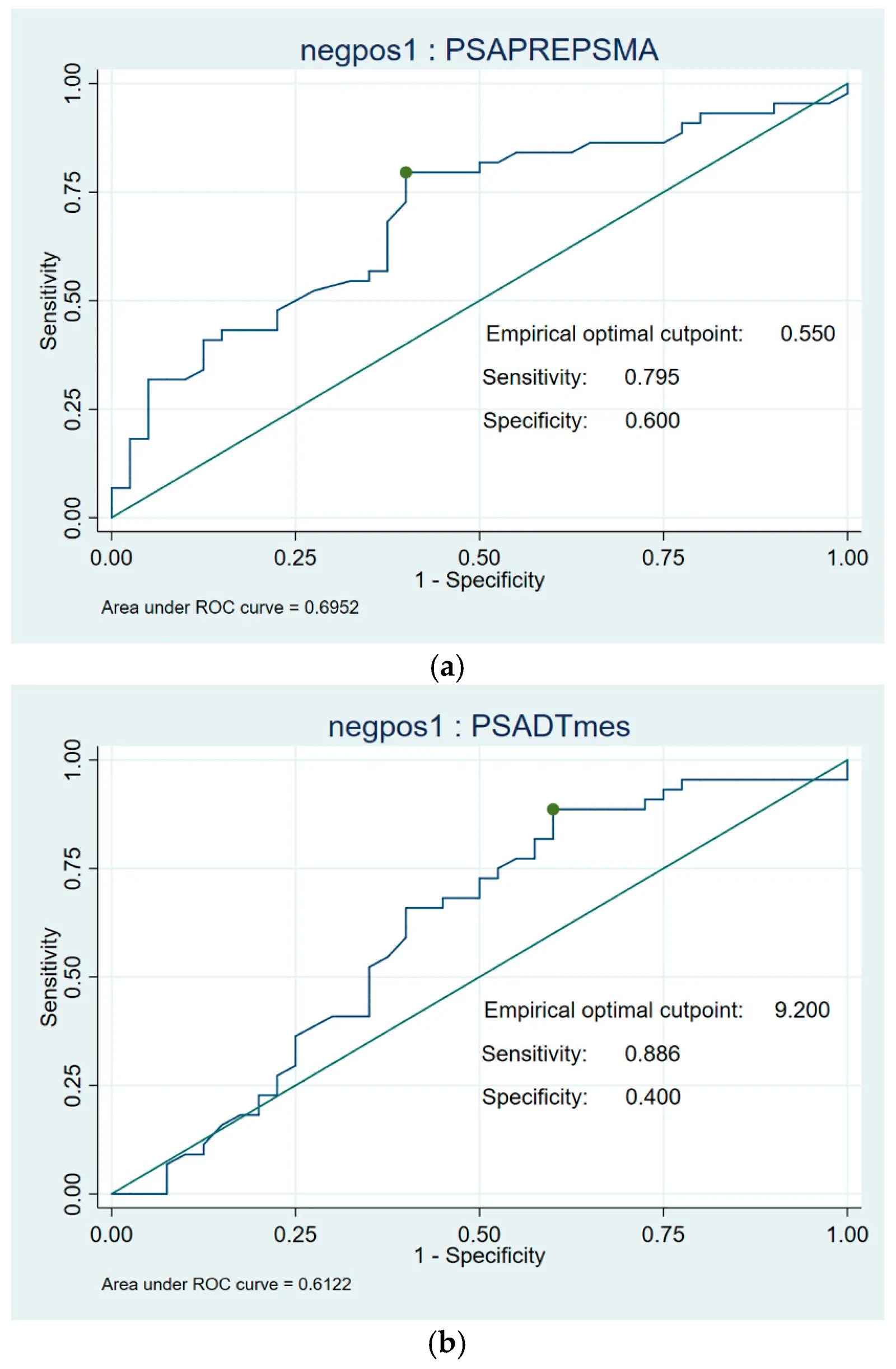
ROC curves for (**a**) PSA (ng/mL) and (**b**) PSA-DT (months) in discriminating between positive and negative PET/CT findings.

These findings were further characterized by ROC analysis, which identified a cohort-derived PSA threshold of 0.55 ng/mL for discriminating between positive and negative PET findings (sensitivity, 84%; specificity, 60%; AUC, 0.69), while a PSA-DT threshold of 9.2 months showed more limited discriminatory performance (sensitivity, 89%; specificity, 37%; AUC, 0.61), suggesting greater discriminatory ability for PSA than for PSA kinetics in this cohort (Figure 2).

### 3.2. Treatment Intensification and Changes in Radiotherapy Planning

A total of 85 patients with BCR of PC were evaluated for salvage treatment strategies prior to undergoing 18F-DCFPyL-PSMA PET/CT. The original treatment intention was defined based on clinical parameters before PET imaging. Among patients without prior radiotherapy, 47.1% (40/85) were considered candidates for SRT to the PB, with or without ADT depending on the presence of high-risk features and previous exposure to ADT. An additional 27% (23/85) had previously received ART or SRT without ADT. In this subgroup, the initial management strategy consisted of initiating ADT alone. The remaining 25.9% (22/85) had undergone prior multimodal treatment, including ART, SRT, and ADT. Within this heavily pretreated group, 17 patients were initially assigned to active surveillance with close PSA monitoring, while 5 were considered for reinitiation of ADT.

Overall, changes from the original treatment intention to the post–18F-DCFPyL-PSMA PET/CT treatment intention were observed in 97.8% (44/45) of patients with positive findings and in 52.5% of those with negative findings (*p* < 0.001).

**Figure 3:**
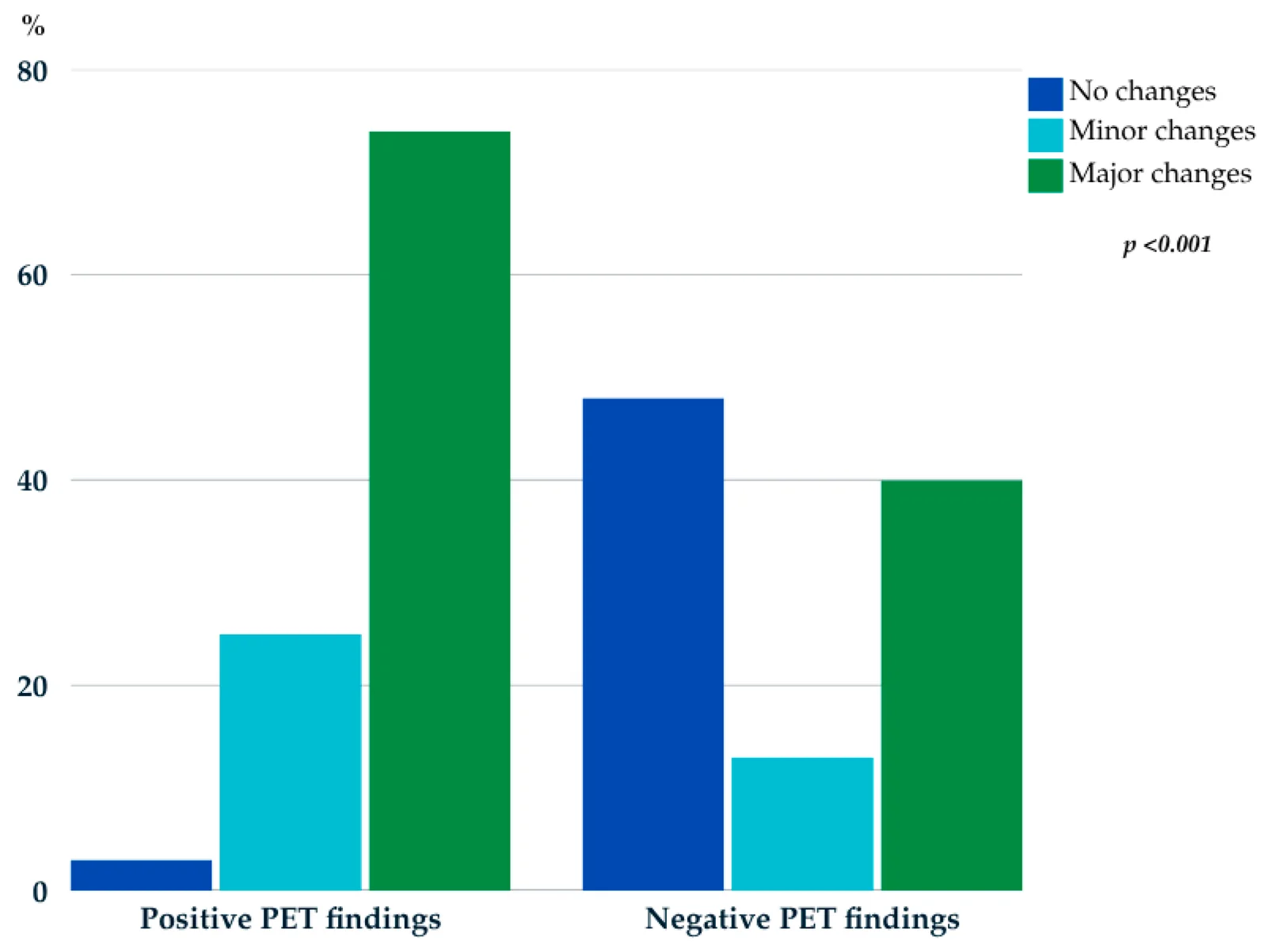
Treatment changes according to 18F-DCFPyL PSMA PET/CT results.

**Figure 4:**
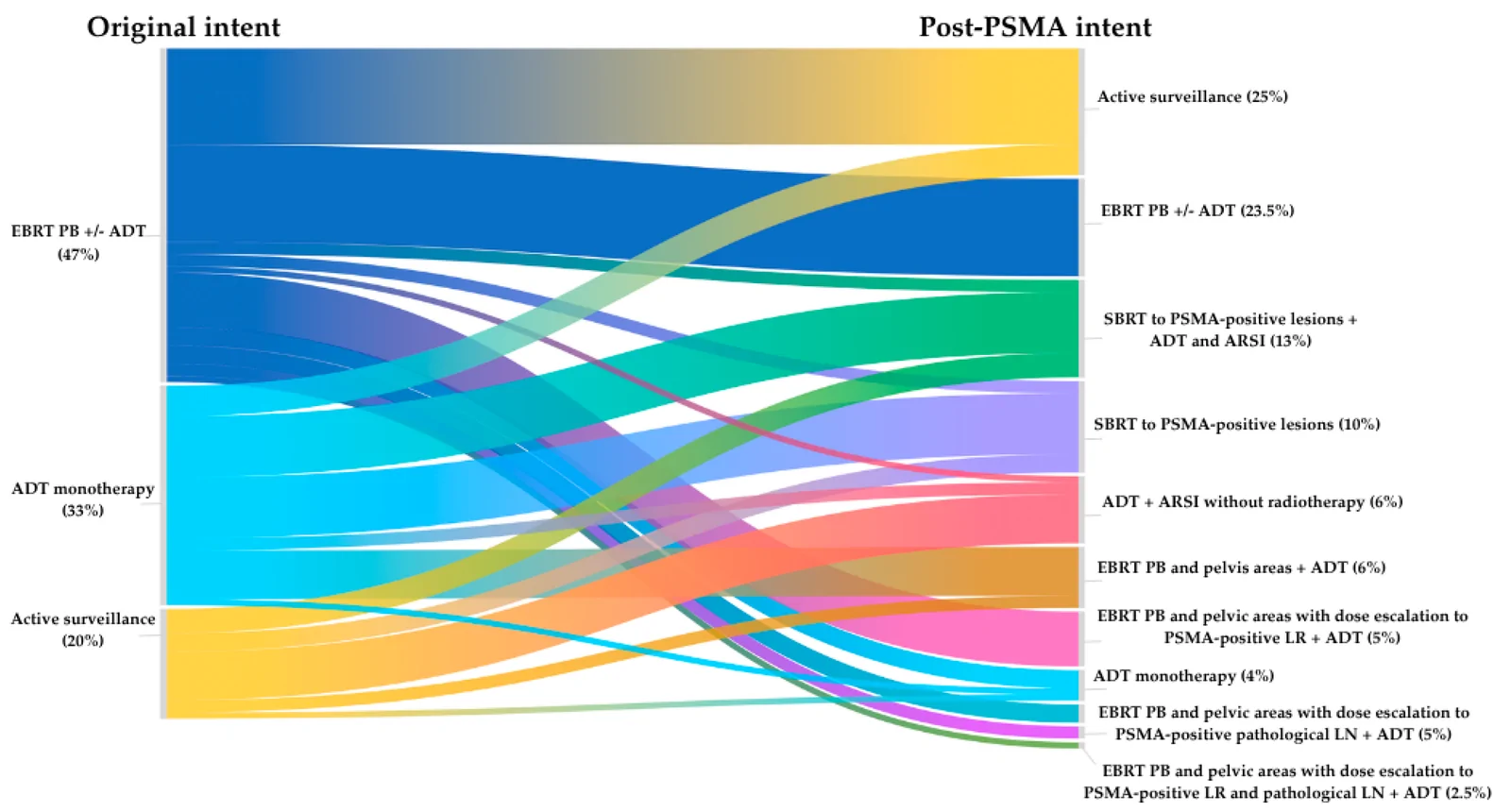
Sankey diagram illustrating patient-level changes in treatment strategy from initial intent to post–18F-DCFPyL PSMA PET/CT intent, including both PET-positive and PET-negative cases. The diagram depicts the redistribution of patients across therapeutic approaches following PET imaging, including shifts toward locoregional radiotherapy intensification, metastasis-directed therapy with SBRT, systemic treatment intensification, and in selected cases de-escalation to active surveillance.

In patients with negative PET findings, 47% showed no change from the original treatment intent, corresponding to individuals without prior radiotherapy who proceeded with PB radiotherapy, of whom 7.7% received additional ADT. Minor changes were observed in 13% of cases, and consisted of radiotherapy planning modifications, including expansion to EPRT combined with ADT. Major changes occurred in 40% of patients, in whom negative PET findings supported a shift toward active surveillance with close PSA monitoring, without immediate local or systemic treatment. In contrast, among patients with positive PET findings, only 2.2% maintained the original treatment plan (PB radiotherapy plus ADT). Minor changes were observed in 24.5% of cases, and primarily consisted of local or locoregional radiotherapy adaptations, including dose escalation to PSMA-positive lesions within areas of LR in the PB or in pelvic LNs. Major treatment changes were identified in 73.3% of patients, and were mainly driven by the detection of oligometastatic disease, prompting a shift toward metastasis-directed therapy with SBRT, with or without systemic treatment intensification (ADT plus ARSI). In a minority of cases (9%), active surveillance was recommended (Figure 3 and Figure 4).

Changes in radiotherapy planning, as summarized in Table 2, were observed in 84.4% (38/45) of cases with positive PET findings. The main impact was an increased recommendation for treatment intensification, reflecting a shift toward more personalized strategies guided by disease distribution. Locoregional treatment intensification with external beam radiotherapy (EBRT) to the PB, with or without PRT and dose escalation to PSMA-positive LR in the PB or pelvic LNs, combined with ADT, was performed in 18.3% of patients. Additionally, 10.5% of patients received PRT with dose escalation to PSMA-positive LNs in combination with ADT. In contrast, 50% of patients underwent metastasis-directed therapy with SBRT targeting PSMA-positive lesions, with 29% combined with systemic treatment intensification using ADT plus ARSI. Systemic treatment alone was selected in 21.2% of patients, including ADT with ARSI (13.2%) or ADT monotherapy (8%).

**Figure 5:**
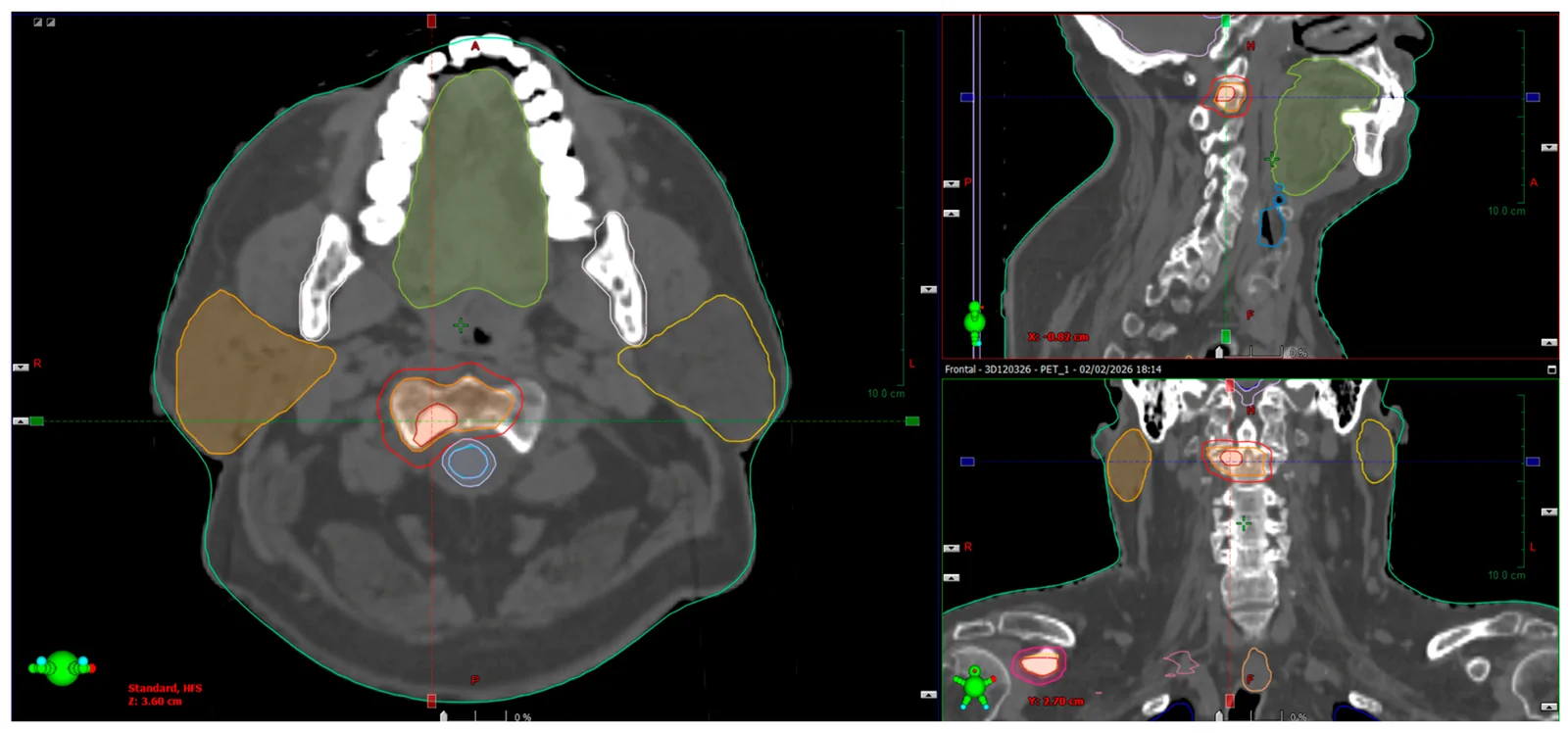
Treatment planning of SBRT directed to a PSMA-positive lesion in the second cervical vertebra (C2), administered in combination with ADT and ARSI.

**Figure 6:**
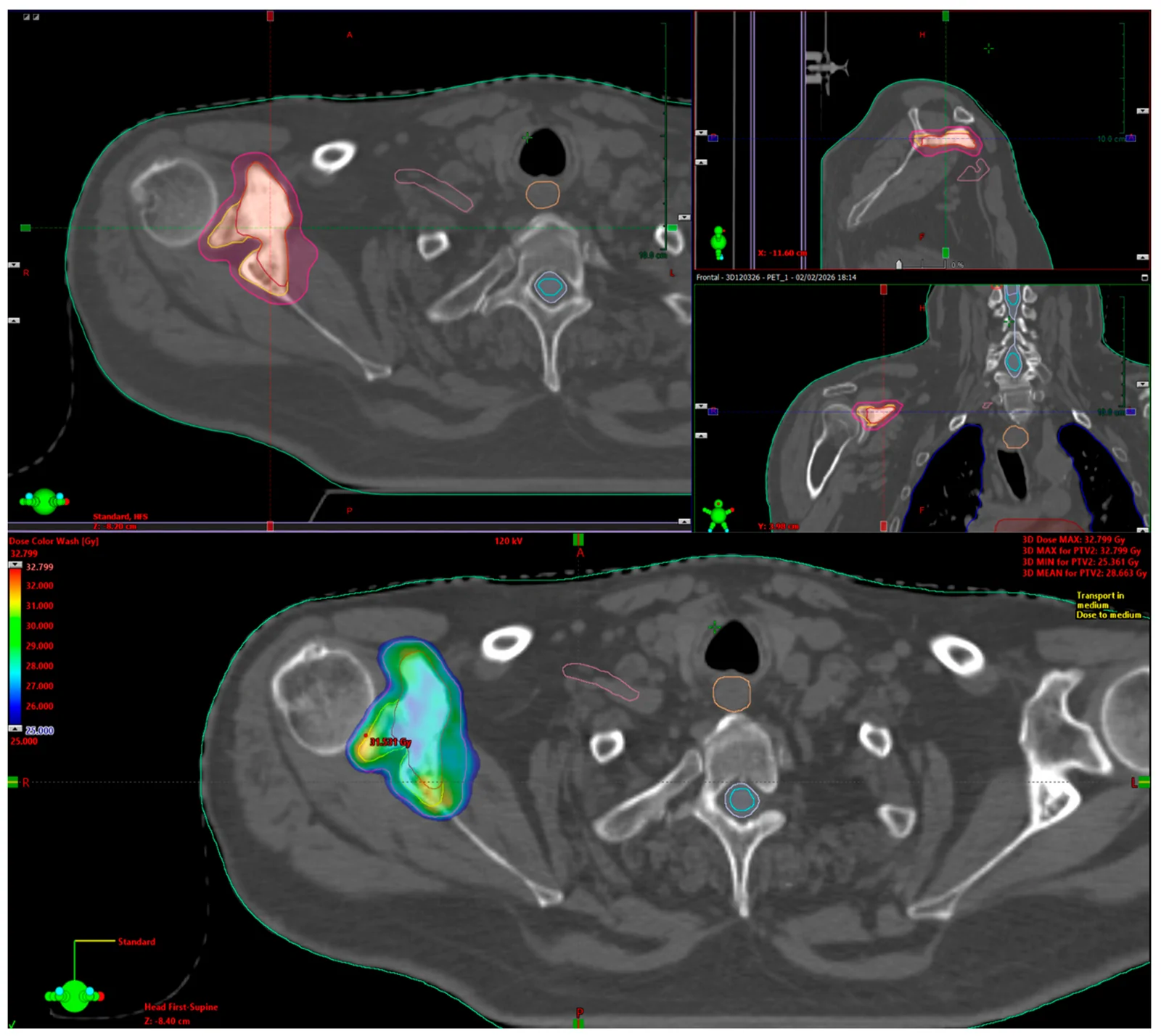
Treatment planning and dose distribution of SBRT directed to a PSMA-positive lesion in the coracoid process of the right scapula.

**Figure 7:**
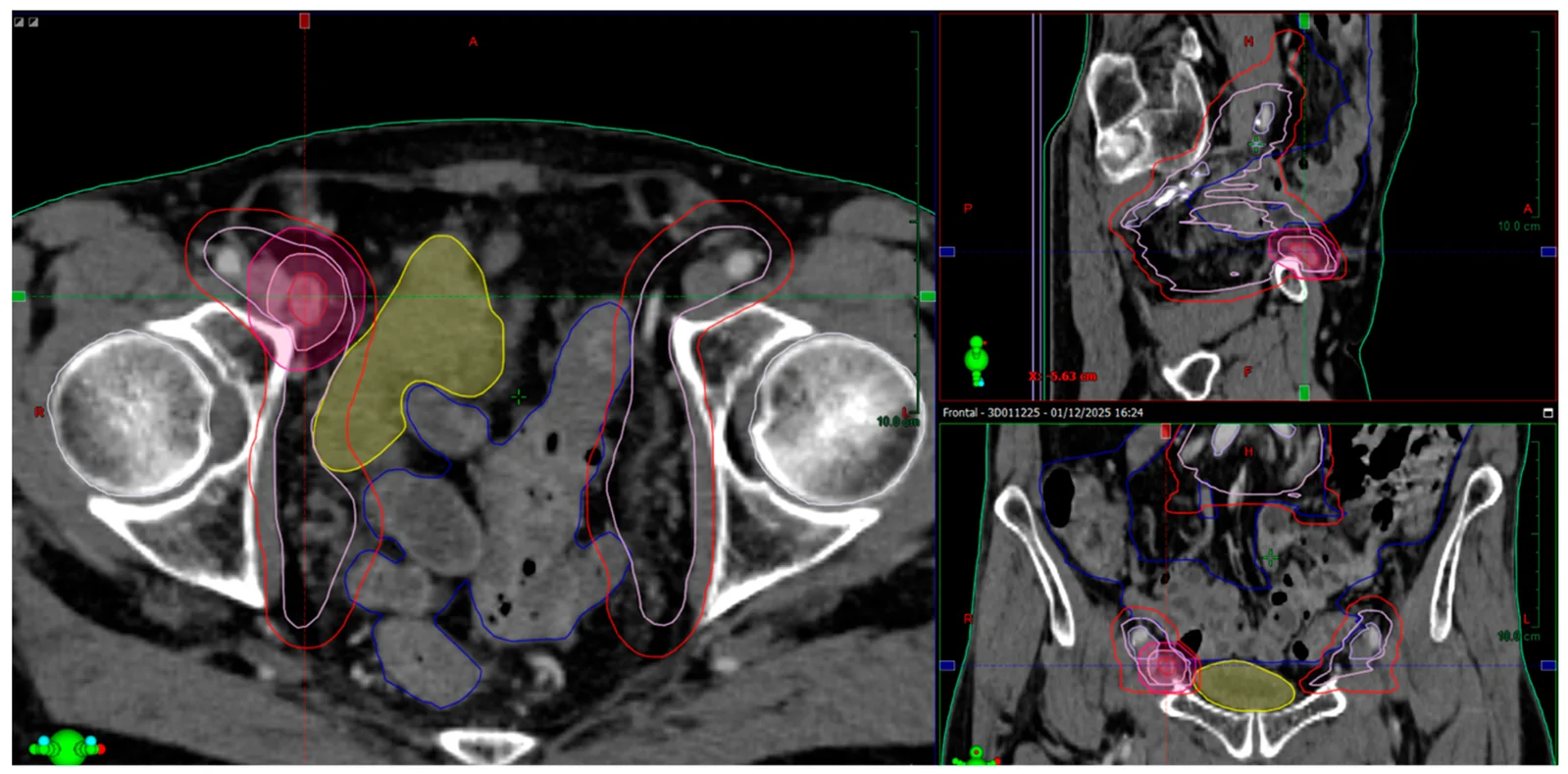
Pelvic radiotherapy with dose escalation to a PSMA-positive pathological lymph node.

The most commonly used SBRT regimen consisted of 24–27 Gy delivered in three fractions (8–9 Gy per fraction), provided that organ-at-risk (OAR) constraints were met. PRT was delivered to 45–46 Gy in 1.8–2.0 Gy fractions, with concomitant dose escalation up to 55–56 Gy in cases of pathological LN involvement. PB radiotherapy was administered at 66–68 Gy in 33–34 fractions (2 Gy per fraction), with dose escalation up to 70–72 Gy in PSMA-positive areas. Overall, these findings indicate that 18F-DCFPyL-PSMA PET/CT substantially influenced radiotherapy target volumes and therapeutic decision-making, including the selection of metastasis-directed strategies and systemic treatment intensification in a substantial proportion of patients (Figure 5, Figure 6 and Figure 7).

After implementing the changes in therapeutic approach based on PSMA PET/CT findings and following a 24-month follow-up period, the median PSA level was 0.08 ng/mL (*p* < 0.001).

The following categories represent the time required to achieve a BR, defined as a reduction of ≥50% from the pretreatment PSA level, rather than the total duration of follow-up. Among patients with positive PET findings, 35.6% achieved a BR within the first 3 months following treatment adaptation, 46.7% between 3 and 6 months, and 6.7% after 6 months. Overall, 11% of patients did not achieve a BR during follow-up. These cases included slow biochemical progression (4.5%), early bone or nodal progression (4.5%), and progression to castration-resistant prostate cancer (2%). Notably, early progression was predominantly observed in patients who did not undergo treatment modification despite positive PET findings, including one patient who rapidly developed castration-resistant disease within 3 months. BRs were observed most frequently within the first 6 months among PET-positive patients who underwent major treatment modifications; however, these descriptive findings do not allow the effects of individual treatment components to be distinguished.

**Figure 8:**
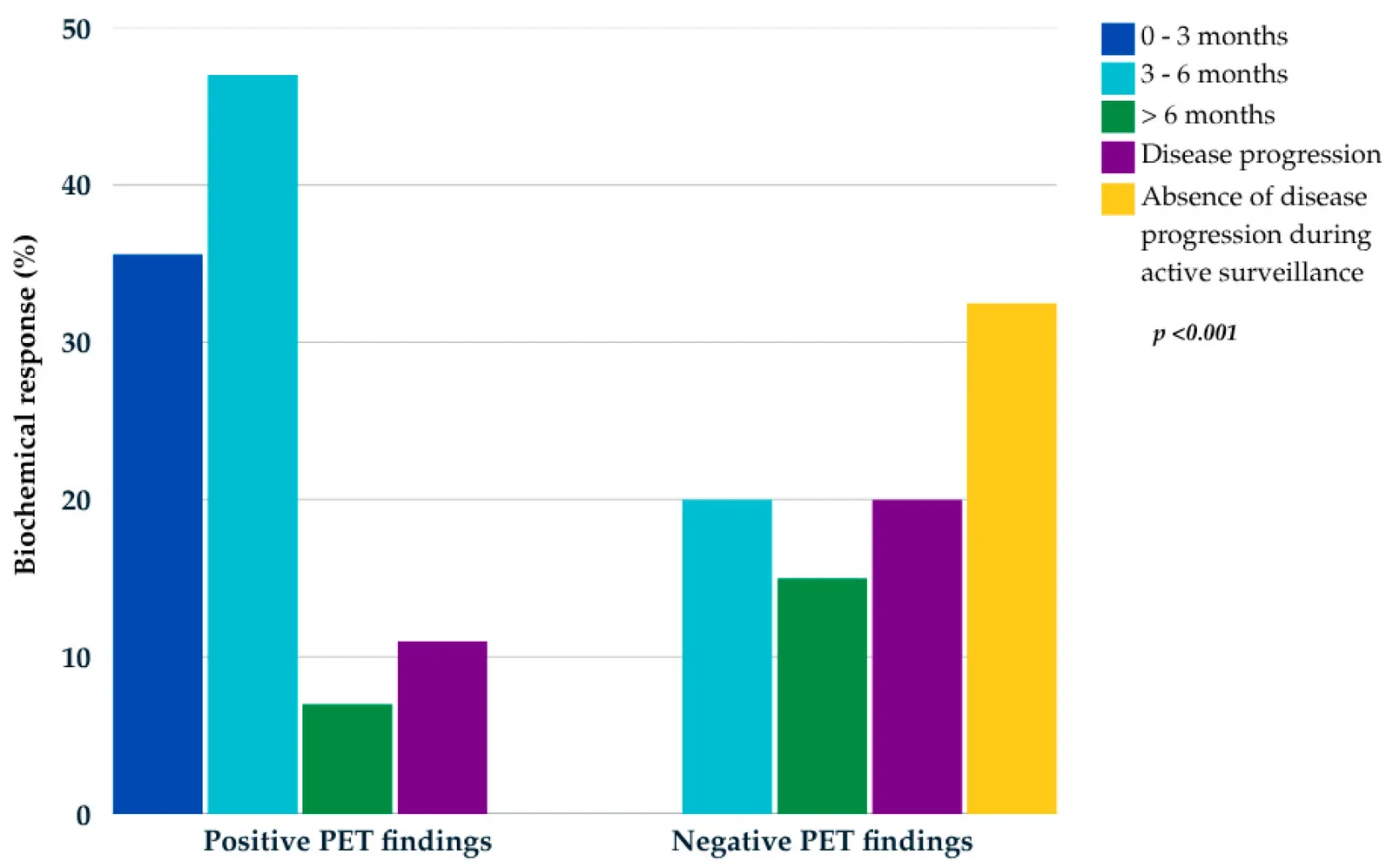
Biochemical response kinetics and progression patterns stratified by 18F-DCFPyL-PSMA PET/CT findings.

In the cohort of patients with negative PET findings, 19 maintained the original treatment intent. Within this group, 20% achieved a BR between 3 and 6 months and 15% after 6 months. Disease progression was observed in 12.5% (five patients), including three cases following PB radiotherapy and two during ongoing ADT. Minor treatment modifications were performed in five patients, all of whom achieved a BR after 6 months. Among patients in whom negative PET findings led to a strategy of active surveillance, 32.5% remained free from disease progression during follow-up, while 7.5% developed progression (Figure 8).

Overall, these findings suggest that 18F-DCFPyL-PSMA PET/CT-guided treatment adaptation, particularly when involving major therapeutic changes, was associated with higher rates of early BR within the study cohort, whereas early disease progression was more frequently observed among patients whose treatment strategy remained unchanged, particularly in those with positive PET findings.

## 4. Discussion

PSMA-targeted radiotracers have become a cornerstone in molecular imaging for PC [52,53], with several ligands demonstrating high affinity for the extracellular domain of PSMA and consistent diagnostic performance across different disease settings [54,55,56]. Among these, 68Ga-PSMA-11, 18F-DCFPyL-PSMA, and 18F-PSMA-1007 are the most widely used in clinical practice and research and are collectively referred to as PSMA ligands due to their similar biological behavior [31,57,58]. 68Ga-PSMA-11 has the longest history of clinical use and the strongest scientific evidence supporting its efficacy [59,60,61,62].

Notably, 18F-DCFPyL has demonstrated a disease DR comparable to that of 68Ga-PSMA-11 without an increase in false-positive findings, and in some series even higher DR, which may be attributed to the superior spatial resolution and imaging characteristics of 18F-labeled tracers [30,63].

In this context, conventional imaging modalities such as abdominopelvic CT, pelvic MRI, and bone scintigraphy remain limited by their relatively low sensitivity for detecting occult BCR. Although PET radiotracers such as 18F/11C-choline and 18F-fluciclovine were previously established in this setting, their use has declined with the emergence of PSMA-targeted tracers. PET/CT with PSMA ligands has demonstrated superior sensitivity and specificity for detecting both local and distant disease, particularly in early recurrence of PC at low PSA levels [22,34,36,61]. Beyond the BCR setting, PSMA PET has also shown higher accuracy than conventional imaging for nodal and bone staging, as well as greater sensitivity than multiparametric MRI for local tumor assessment when combined with MRI [64]. Accordingly, current international guidelines recommend PSMA PET/CT for the evaluation of occult BCR, although the optimal PSA threshold for its use remains to be clearly defined.

Our prospective observational study of 85 patients demonstrated the significant impact of 18F-DCFPyL-PSMA PET/CT on clinical decision-making in patients with BCR of PC following RP with negative or nonspecific findings on conventional imaging. Our findings indicate that PSMA PET/CT not only provides additional disease detection beyond conventional imaging, but more importantly leads to substantial modifications in treatment strategy, with a particularly high impact on radiotherapy planning. Molecular imaging facilitates the localization of recurrent disease and informs individualized treatment planning. In line with this, current EAU guidelines recommend PSMA-targeted PET/CT in patients with BCR after RP, particularly at PSA levels of 0.2–0.5 ng/mL, when the results are expected to influence subsequent therapeutic decisions [38,39,40,65].

Our findings demonstrate a robust DR across varying PSA levels and PSA-DT, supporting the ability of 18F-DCFPyL-PSMA PET/CT to identify both local and distant disease. DRs increased significantly with higher PSA values, reaching 31.3%, 60%, and 77.8% for PSA levels 0.2–0.5, 0.5–0.9, and 1.0 ng/mL, respectively, highlighting the reliability of this imaging modality even at relatively low PSA levels. Similarly, higher DRs were also observed among patients with shorter PSA-DT, reaching 61.5% for PSA-DT < 6 months and 50% for PSA-DT between 6 and 12 months. In addition, DRs increased with tumor aggressiveness, with values of 24%, 59%, and 68.4% for ISUP 1–2, 3, and 4–5, respectively. Overall, these findings indicate a positive association between higher PSA levels and shorter PSA-DT with PSMA PET positivity, reflecting the combined influence of tumor burden and biological aggressiveness. Notably, most patients in our cohort presented with high-risk disease (Gleason score ≥ 8, ISUP 4–5), which may have contributed to the relatively high DRs observed.

These results are consistent with findings from pivotal studies and further reinforce the critical role of PSMA PET/CT in the management of BCR of PC. The diagnostic performance of 18F-DCFPyL-PSMA PET/CT has been well established in prospective trials such as the CONDOR trial (Morris et al.) [66], a phase III trial including 208 patients with negative conventional imaging that demonstrated clinically meaningful DRs even at low PSA levels, ranging from 36.2% for PSA < 0.5 ng/mL to 96.7% for PSA ≥ 5 ng/mL, with a corresponding impact on clinical management in 63.9% of cases. Similarly, Rousseau et al. [67] reported high DRs across different PSA strata, reaching 60% for PSA levels of 0.4–0.5 ng/mL, 78% for PSA 0.5–1.0 ng/mL, and up to 92% for PSA ≥ 2.0 ng/mL, further confirming the strong dependence of detection performance on PSA levels. In line with these findings, Fendler et al. [68] and Aydin et al. [69] have highlighted the ability of PSMA PET/CT to detect disease not identified on conventional imaging, including lesions located outside standard radiotherapy fields, even at low PSA levels. These observations are further supported by systematic reviews and meta-analyses. Treglia et al. [70] reported pooled DRs of 86% for PSA ≥ 0.5 ng/mL and 49% for PSA <0.5 ng/mL, while large multicenter data from Afshar-Oromieh et al. [71], including 2533 patients, demonstrated a progressive increase in DRs from 43% for PSA ≤ 0.2 ng/mL to 93% for PSA >10 ng/mL. Importantly, despite its high diagnostic performance, PSMA PET/CT is not without limitations. Its sensitivity decreases in the setting of microscopic disease, particularly at low PSA levels, as reflected by the proportion of negative scans and unchanged management observed in our cohort. In addition, the lack of histopathological confirmation and limited long-term outcome data restrict the ability to fully determine its impact on survival endpoints.

ROC curve analysis in our cohort identified a cohort-derived PSA threshold of 0.55 ng/mL and a PSA-DT threshold of 9.2 months for discriminating between positive and negative PET/CT findings. These findings are in line with those reported by Hoffman et al. [72], who identified a higher PSA threshold of 1.24 ng/mL as the optimal cutoff for predicting positive scan results.

Importantly, beyond these associations with DR, 18F-DCFPyL-PSMA PET/CT enabled the identification of 79 PSMA-positive lesions in our study that were undetectable or nonspecific on conventional imaging. The clinical relevance of these findings was reflected in their direct impact on radiotherapy target definition and therapeutic decision-making, which constituted the principal focus of the present study. Nodal involvement represented the most frequent site of disease (60.7%), followed by bone lesions (22.8%) and LR in the PB (12.7%), while visceral involvement was uncommon and largely restricted to patients with higher PSA levels (3.8%).

These findings are consistent with previous reports demonstrating the superior sensitivity of PSMA-targeted PET/CT compared to conventional imaging. Rowe et al. [73] reported a markedly higher lesion DR with 18F-DCFPyL PET/CT, identifying a substantial number of lesions that were occult or indeterminate on standard imaging modalities, particularly in nodal and osseous sites. Similarly, the OSPREY trial (Pienta and Gorin et al.) [36], a pivotal phase II/III study, confirmed the high diagnostic accuracy of 18F-DCFPyL PET/CT, demonstrating its ability to detect previously unsuspected disease in the PB, LNs, and distant sites, with reported sensitivity of up to 89% even at low PSA levels. Roach et al. [62], in a multicenter study including 431 patients, reported management changes in up to 51% of cases, increasing to 62% in the BCR setting, largely driven by the detection of previously unrecognized disease in the prostate bed, lymph nodes, and distant metastatic sites. Taken together, these published data, along with our findings, highlight the ability of PSMA PET/CT to enhance disease characterization and inform more personalized, risk-adapted therapeutic strategies.

Beyond its diagnostic performance, PSMA PET/CT can substantially influence salvage treatment planning by guiding radiotherapy field adaptation and informing treatment intensification. In our cohort, prior to undergoing 18F-DCFPyL PSMA PET/CT, all patients were evaluated for salvage treatment strategies. Patients without prior radiotherapy were considered for radiotherapy to the PB, with the addition of ADT in the presence of high-risk features [51]. In contrast, for patients who had already received radiotherapy, management options included ADT alone or active surveillance. This approach was defined as the original treatment intent. Although several randomized trials support the use of adjuvant or salvage radiotherapy in patients with BCR after RP, more than 15–20% of patients in these studies failed to achieve biochemical control [8,11,74,75,76,77]. This likely reflects the presence of disease beyond the PB, highlighting the need for more sensitive imaging techniques to accurately localize recurrent disease in this clinical setting, where PSMA ligand PET/CT has emerged as a key diagnostic tool in recent years [36,71,72,78].

In our cohort, positive 18F-DCFPyL-PSMA PET/CT findings had a profound impact on clinical decision-making, leading to treatment modifications in most patients. Minor changes were observed in 24.5% of PET-positive cases and were primarily related to radiotherapy planning adjustments, including dose escalation to PSMA-positive lesions and expansion of target volumes to encompass previously unrecognized disease. In contrast, major changes were observed in 73.3% of cases and were predominantly driven by the detection of oligometastatic disease, prompting a shift toward metastasis-directed therapy with SBRT, frequently combined with systemic treatment intensification using ADT and ARSIs. Conversely, negative PET findings were more often associated with treatment de-escalation, supporting the adoption of active surveillance in selected patients.

Minor changes preserve the original treatment intent by modifying radiotherapy fields through target volume expansion or dose escalation to PSMA-positive lesions. Studies such as those by Frenzel et al. [46] and Karagiannis et al. [79] have shown that the higher sensitivity of PSMA PET/CT translates into clinically relevant modifications of radiotherapy fields in up to 46% and 50% of cases, respectively, primarily through the identification of additional lesions not detected on conventional imaging. This often results in treatment intensification, including expansion of target volumes or delivery of focal dose escalation to PSMA-positive lesions, as well as treatment de-escalation when no disease is identified in selected cases. Similarly, Calais et al. [80] demonstrated that PSMA PET/CT can significantly refine radiotherapy target delineation, with PSMA-positive lesions located outside conventional CTVs in 16.5% of patients. This proportion increased to 32% when pelvic LNs were not initially included. Notably, a proportion of pelvic nodal metastases, particularly in the perirectal and common iliac regions, would have been systematically missed using standard contouring guidelines, highlighting the limitations of anatomy-based planning. These observations are further supported by Onal et al. [81], who reported that the integration of 68Ga-PSMA PET/CT into radiotherapy planning led to meaningful modifications of treatment fields in approximately 13% of patients, mainly driven by the detection of additional pelvic LN involvement or distant metastatic sites not identified on conventional imaging. Likewise, Schmidt-Hegemann et al. [82] reported modifications in radiotherapy management in 56.6% of patients, primarily through target volume expansion, incorporation of previously unrecognized nodal or distant disease, and the use of simultaneous integrated boost strategies.

Overall, these data reinforce the role of PSMA PET/CT in shifting radiotherapy planning from empirical field design toward a more individualized, biology-driven approach and support its value in informing disease-adapted radiotherapy planning. These findings are consistent with our results and support the use of PSMA PET/CT as a key modality for enabling more personalized, disease-adapted treatment strategies.

Regarding major treatment changes, PSMA PET/CT plays a pivotal role in identifying oligometastatic disease, particularly in LN and bone disease, thereby enabling a shift toward metastasis-directed strategies. In this context, patients may be candidates for high-dose lesion-directed radiotherapy using SBRT with or without systemic treatment intensification. Importantly, previous studies suggest that improved detection of distant metastases in the setting of BCR may help avoid unnecessary PB radiotherapy in selected patients, with potential implications for treatment-related toxicity and healthcare resource use. In addition, metastasis-directed treatment of oligometastatic disease has been associated with improved progression-free outcomes and delayed initiation of ADT in prospective studies [83].

The role of metastasis-directed radiotherapy in this setting has been evaluated in several prospective trials. In the phase II randomized ORIOLE study, Phillips et al. [84] demonstrated that SBRT significantly reduced disease progression compared with observation (19% vs. 61% at 6 months), with a marked improvement in progression-free survival. Notably, complete consolidation of PSMA-avid disease was associated with a significantly lower risk of new metastases, underscoring the importance of PSMA-guided treatment strategies. SBRT was also well tolerated, with no grade ≥ 3 toxicities reported. Complementary evidence from Henkenberens et al. [85] showed that PSMA-guided SBRT in castration-resistant disease achieved significant biochemical responses, prolonged biochemical progression-free survival, and delayed the need for second-line systemic therapies. Similarly, Kneebone et al. [86] reported excellent local control with no in-field failures and favorable biochemical outcomes, further supporting the efficacy of this approach. In line with these findings, Koerber et al. [87] demonstrated high biochemical response rates exceeding 80% and encouraging long-term outcomes, with a 3-year biochemical progression-free survival of 55%. However, the occurrence of new lesions outside treated sites highlights the importance of comprehensive disease detection and complete consolidation strategies. Moreover, improved outcomes observed with concomitant ADT or more extensive irradiation approaches suggest a synergistic role for combined local and systemic treatment strategies.

Collectively, these published data support the role of metastasis-directed therapy in selected patients with oligometastatic PC and the integration of PSMA PET/CT into treatment planning to inform metastasis-directed treatment strategies, as reflected in current international consensus statements [88].

Extending this concept beyond its role in guiding EBRT, PSMA PET/CT is also a central component of the evolving theranostic approach to PC. In patients with a higher metastatic burden or more disseminated disease, assessment of PSMA expression and distribution may identify candidates for PSMA-targeted radioligand therapy, thereby linking molecular imaging directly with systemic tumor-directed radiation delivery. 177Lu-PSMA-617 has demonstrated clinical benefit in appropriately selected patients with PSMA-positive metastatic castration-resistant PC, for whom diagnostic PSMA PET/CT is essential to confirm sufficient target expression and exclude clinically relevant PSMA-negative disease [89,90]. PSMA-targeted alpha therapy using 225Ac-PSMA may represent a further therapeutic approach; however, it remains investigational, and its efficacy, optimal sequencing, and toxicity profile require further prospective evaluation [90,91]. Although these theranostic applications are primarily relevant to more advanced disease settings than the early-BCR population evaluated in the present study, they illustrate the broader role of PSMA PET/CT in informing therapeutic selection across the PC disease continuum.

In summary, our findings support the clinical utility of 18F-DCFPyL-PSMA PET/CT for detecting recurrent disease in patients with BCR of PC. Beyond its diagnostic value, PSMA PET/CT provides clinically relevant information that informs therapeutic decision-making, including treatment intensification or de-escalation, and supports individualized, disease-adapted treatment planning. Together, these results reinforce its role as a tool for guiding a more precise, biology-driven approach to salvage treatment planning.

### Limitations and Future Directions

Our study has some limitations that should be acknowledged. First, although designed as a prospective observational study, the sample remains relatively small. In addition, long-term outcome data, including progression-free survival (PFS) and overall survival (OS), are not yet available, as these were not primary endpoints of the current analysis. Moreover, the single-arm observational design and the absence of a comparator group managed without PSMA PET/CT preclude establishing a causal relationship between PSMA-guided treatment modifications and the observed biochemical responses. Therefore, these findings should be interpreted as exploratory associations rather than as evidence of treatment efficacy, since biochemical response may also have been influenced by treatment modality, systemic therapy use, and underlying disease characteristics. The cohort also included clinically distinct scenarios with heterogeneous prior treatment exposures, particularly regarding RT and ADT. Although this reflects real-world clinical practice and pre-PET treatment intent was defined according to each patient’s treatment history, this heterogeneity should be considered when interpreting the pooled results. Furthermore, no direct comparison between PSMA PET/CT and conventional imaging modalities was performed, as all included patients had negative or inconclusive findings on standard imaging; thus, the primary aim was to evaluate the added value of metabolic imaging in this specific clinical scenario. Histopathological confirmation of PSMA PET/CT-positive lesions was not systematically performed, which should also be considered when interpreting the reported detection findings. The present sample size was not considered sufficient for the development and internal validation of a robust multivariable model of PET positivity. Ongoing efforts are focused on expanding the study cohort, particularly in patients with low PSA levels, and on conducting dedicated analyses comparing PSMA-guided radiotherapy planning with conventional imaging–based approaches. Additionally, future analyses based on an expanded cohort, longer follow-up, and a prespecified modeling strategy will evaluate whether a clinically useful and internally validated pre-PET model can be developed. Future work will include comparative studies of different 18F-labeled PSMA tracers to further refine imaging strategies in this setting.

## 5. Conclusions

18F-DCFPyL-PSMA PET/CT provided clinically relevant imaging information for the evaluation of patients with PC and occult BCR after RP. In our cohort, it demonstrated robust DRs even at low PSA levels and more importantly led to substantial modifications in therapeutic management, particularly in radiotherapy planning and treatment intensification. The integration of PSMA PET/CT informed both treatment escalation through the identification of locoregional and oligometastatic disease and de-escalation strategies in selected patients with negative findings, supporting a more individualized treatment paradigm. Notably, treatment strategies were modified in most patients with positive imaging, underscoring the strong influence of molecular imaging on clinical decision-making.

Overall, our findings highlight the dual diagnostic and treatment planning value of 18F-DCFPyL-PSMA PET/CT, supporting its role as a key tool for informing individualized, biology-driven salvage treatment planning in patients with BCR of PC.

## Figures and Tables

**Table 1:** Patient characteristics (*n* = 85).

Characteristic	Value
Age	48–74 years
Median age	69 years
Mean diagnosis PSA	11.24 ng/mL
TNM	
T2 a–b	7
T2c	36
T3a	21
T3b	20
T4	1
Nx	32
N0	49
N1	4
Mx	32
M0	53
Histological characteristics	
Gleason score < 8	37
Gleason score ≥ 8	48
ISUP 1–2	25
ISUP 3	22
ISUP 4–5	38
Primary treatment	
RP	41
PR + PLND	44
Radiotherapy	
No	40
Adjuvant	12
Salvage	33
ADT	
Yes	33
No	52
Median PSA (pre-DCFPyL PET/CT)	0.59 ng/mL (0.29–1.0)
Mean PSA-DT (pre-DCFPyL PET/CT)	7.2 months (3–17.1)

**Table 2:** Post–18F-DCFPyL PSMA PET/CT treatment strategies in patients with PET-positive findings and treatment modification.

Post-PSMA Treatment Category	Patients (%)
Locoregional intensification	
EBRT to PB with dose escalation to PSMA-positive LR plus ADT	2.6
EBRT to PB and elective pelvic areas with dose escalation to PSMA-positive LR plus ADT	10.5
EBRT to PB and pelvic areas with dose escalation to PSMA-positive LR and pathological pelvic LN plus ADT	5.2
EBRT to pelvic areas with dose escalation to PSMA-positive pathological pelvic LN plus ADT	10.5
Metastasis-directed therapy	
SBRT to PSMA-positive lesions	21
SBRT to PSMA-positive lesions combined with ADT and ARSI	29
Systemic therapy alone	
ADT plus ARSI without radiotherapy	13.2
ADT monotherapy (continuous or intermittent)	8

## Data Availability

The original contributions presented in this study are included in the article. Further inquiries can be directed to the corresponding author.

## Abbreviations

The following abbreviations are used in this manuscript.

BCR	biochemical recurrence
PC	prostate cancer
RP	radical prostatectomy
PSA	prostate-specific antigen
SRT	salvage radiotherapy
ART	adjuvant radiotherapy
PB	prostate bed
CT	computed tomography
MRI	magnetic resonance imaging
PET/CT	positron emission tomography/computed tomography
PSMA	prostate-specific membrane antigen
ISUP	International Society of Urological Pathology
TNM	tumor–node–metastasis
ADT	androgen deprivation therapy
PSA-DT	prostate-specific antigen doubling time
LN	lymph node
DR	detection rate
PRT	pelvic radiotherapy
SBRT	stereotactic body radiation therapy
ARSI	androgen receptor signaling inhibitor
EPRT	elective pelvic radiation therapy
ROC	receiver operating characteristic
PLND	pelvic lymph node dissection
OAR	organs at risk
EBRT	external beam radiotherapy
LR	local recurrence
BR	biochemical response
CTV	clinical target volume
